# Video-assisted thoracic surgery for pleuroperitoneal communication

**DOI:** 10.1186/s40792-019-0595-8

**Published:** 2019-02-19

**Authors:** Shota Mitsuboshi, Hideyuki Maeda, Masato Kanzaki

**Affiliations:** Department of Thoracic Surgery, Tokyo Women’s Medical University, 8-1 Kawada-cho, Shinjuku-ku, Tokyo, 162-8666 Japan

**Keywords:** Pleuroperitoneal communication, Continuous ambulatory dialysis, Video-assisted thoracic surgery

## Abstract

**Background:**

Hydrothorax due to pleuroperitoneal communication (PPC) is a rare complication of continuous ambulatory peritoneal dialysis (CAPD). Approximately 50% of the patients need to convert to hemodialysis.

**Case presentation:**

A 65-year-old man with chronic renal failure due to membranoproliferative glomerulonephritis underwent CAPD. Seven months after starting CAPD, a chest X-ray showed a right hydrothorax. For performing radioscintigraphy, ^99m^Tc-macro-aggregated albumin was administered into the peritoneal cavity with dialysate, and a leakage point of the dialysate into the right pleural cavity was detected. He was diagnosed with PPC and underwent video-assisted thoracic surgery (VATS). The hole was closed by direct suturing and reinforced by a pedicled latissimus dorsi muscle flap (LDM). The patient resumed CAPD 7 weeks later and had no recurrence of the right hydrothorax.

**Conclusions:**

VATS was a useful method for treating PPC. A pedicled LDM flap is an effective material for preventing the recurrence of PPC.

## Background

Hydrothorax due to pleuroperitoneal communication (PPC) is a rare complication of continuous ambulatory peritoneal dialysis (CAPD), and its incidence rate is estimated at approximately 1.6% of all patients undergoing CAPD [1]. Approximately 50% of the patients need to convert to hemodialysis [1]. This article reported a case of video-assisted thoracic surgery (VATS) for PPC.

## Case presentation

**Fig. 1:**
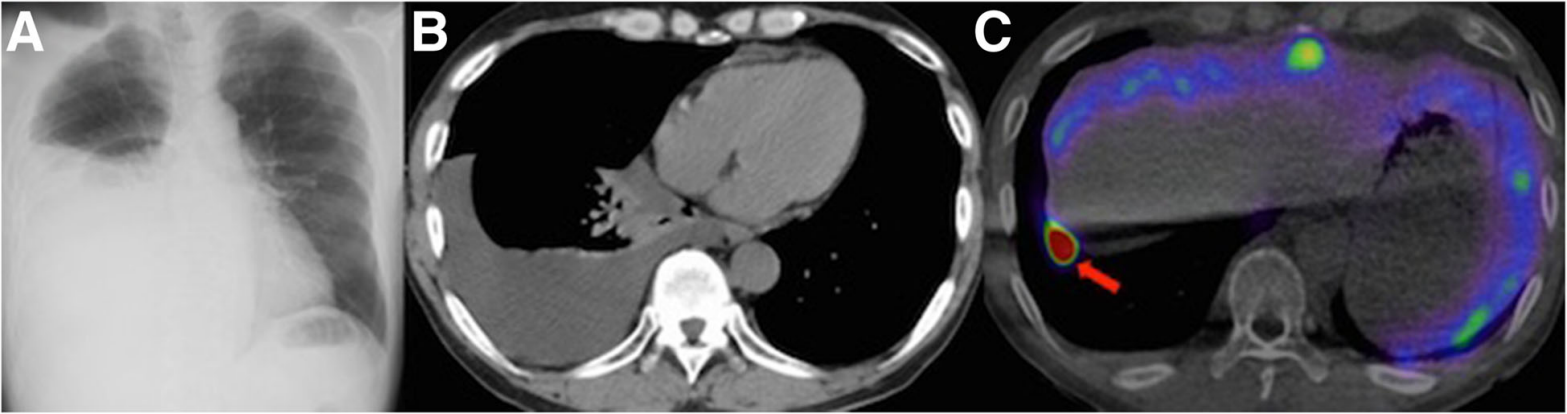
Imaging findings. **a** Chest X-ray radiogram of 65-year-old man with chronic renal failure. Chest X-ray showed a right hydrothorax. **b** Chest computed tomogram (CT) of the patient. Chest CT showed hydrothorax in the right thoracic cavity. **c** Chest ^99m^Tc-macro-aggregated albumin (^99m^Tc-MAA) radioscintigraphic image of the patient. ^99m^Tc-MAA was administered into the peritoneal cavity with dialysate, and 450 min later, a leakage of the dialysate into the right pleural cavity was detected (red arrow)

**Fig. 2:**
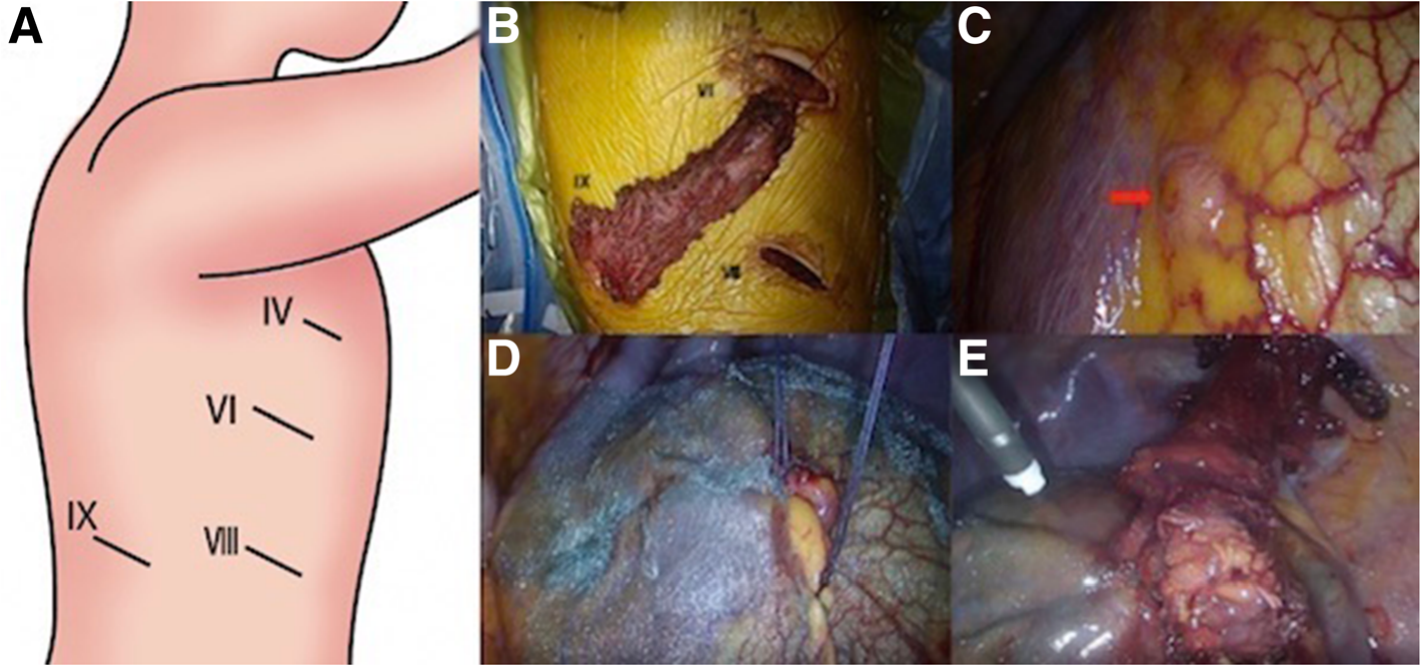
Intraoperative findings. **a** One 2-cm and three 3-cm skin incisions were made at the fourth, sixth, eighth, and ninth intercostal spaces on the posterior axillary lines, respectively. The line marked with “IV” indicates the fourth intercostal space; “VI,” the sixth intercostal space; “VII,” the eighth intercostal space; and “IX,” the ninth intercostal space. **b** The latissimus dorsi muscle (LDM) was accessed and separated from the lower part to the upper along the muscle fiber, and the half of the muscle was eventually harvested as a pedicled LDM flap. **c** A small hole was observed at the right central tendon of the diaphragm (red arrow). **d** The small hole was closed with two 2-0 absorbable multifilament sutures. The central tendon around the reinforcement was covered with a sheet of absorbable polyglycolic-acid felt. **e** The harvested pedicled LDM flap was inserted from the ninth intercostal space of the posterior axillary line. The small hole was reinforced by the harvested pedicled LDM flap. The central tendon around the reinforcement was sprayed with fibrin glue

A 65-year-old man with chronic renal failure due to membranoproliferative glomerulonephritis underwent CAPD. Seven months after starting CAPD, he developed dyspnea. A chest X-ray and computed tomography showed the right hydrothorax (Fig. 1a, b). PPC was suspected; for performing radioscintigraphy for diagnosis, ^99m^Tc-macro-aggregated albumin (^99m^Tc-MAA) was administered into the peritoneal cavity with dialysate, and 240 min later, a leakage point of the dialysate into the right pleural cavity was detected (Fig. 1c). Surgical repair for PPC was planned to resume CAPD. Under general anesthesia, the patient was intubated with a double-lumen endotracheal tube and positioned in a left lateral decubitus position. One 2-cm and three 3-cm skin incisions were made at the fourth, sixth, eighth, and ninth intercostal spaces on the posterior axillary lines, respectively (Fig. 2a). The latissimus dorsi muscle (LDM) was accessed and separated from the lower part to the upper along the muscle fiber, and the half of the muscle was eventually harvested as a pedicled LDM flap (Fig. 2b). A mini-thoracotomy was performed at the eighth intercostal space of the anterior axillary line and ninth intercostal space on the posterior axillary line, XXS-size wound retractors (Alexis^®^ Wound Retractor, Applied Medical, Rancho Santa Margarita, CA, USA) were placed at both places, and a 30°, 10-mm thoracoscope was inserted at the sixth intercostal space of the anterior axillary line. By carefully inspecting the diaphragm with the thoracoscope, the hole was detected at the right central tendon of the diaphragm (Fig. 2c). The lesion was closed with two 2–0 absorbable multifilament sutures (Polysorb^®^, Medtronic, Minneapolis, MN). For avoiding liver damage by suturing, the diaphragm was pulled sufficiently and then sutured. The central tendon around the reinforcement was covered with a sheet of absorbable polyglycolic-acid (PGA) sheet (Neoveil^®^, Gunze, Osaka, Japan) (Fig. 2d). For reinforcing the lesion, the harvested pedicled LDM flap was inserted from the ninth intercostal space of the posterior axillary line and allowed to reach the lesion of the diaphragm. The central tendon around the reinforcement was sprayed with fibrin glue (Beriplast P^®^, CSL Behring, King of Prussia, PA) (Fig. 2e). A 21-Fr silicone drain (silicone thoracic catheter^®^, NIPRO, Osaka) was inserted in the right pleural cavity, and a 15-Fr silicone drain (blake drain^®^, ETHICON, Somerville, NJ) was inserted in the subcutaneous. The chest silicone drain was removed on the fourth postoperative day, because the amount of pleural effusion decreased to less than 100 mL. The subcutaneous silicone drain was removed on the eighth postoperative day, because the amount of drainage decreased. The patient was discharged on the tenth postoperative day. The patient resumed CAPD 7 weeks later, and no recurrence of the right hydrothorax was observed for 14 months.

## Discussion

Hydrothorax due to PPC is a rare but a well-known complication of CAPD [1]. When hydrothorax is found in CAPD patients, the cause is suspected to be PPC. Hydrothorax is usually found in a right-sided cavity and involves cough, dyspnea, and chest pain, but some patients have no symptoms [2].

As the onset cause of PPC, several studies speculate that PPC is speculated to be induced by (1) the defects of diaphragm due to abnormal intraperitoneal pressure, (2) congenital or traumatic diaphragm defects, (3) defects or laceration of blebs in the fragile region of the diaphragm, and (4) migration through lymphatic vessels [3–6]. In this report, the pathogenesis in the patient was suspected to be an abnormal intraperitoneal pressure caused by injecting a CAPD solution, leading to the defect of the diaphragm.

PPC is diagnosed by pleural fluid examination and the transition of ^99m^Tc-MAA radioscintigraphy from peritoneal cavity to pleural cavity. In this case, the preoperative diagnosis of PPC was obtained by using ^99m^Tc-MAA radioscintigraphy.

Although approximately 50% of the patients need to convert to hemodialysis, most of the patients still need to continue CAPD for social reasons [1]. Either a nonsurgical or surgical method is taken for treating PPC. As the nonsurgical method, pleurodesis and the temporary cessation of CAPD are performed. For pleurodesis, OK-432, talc powder, fibrin glue, and minomycin are used. Temporary cessation of CAPD may lead to the spontaneous healing of diaphragmatic defects. However, the nonsurgical method is estimated to be effective for only 50% of all cases [2]. Most of the patients still need to continue CAPD for social reasons, and they require surgical treatment. As the surgical procedure to treat PPC, the resection, direct suturing, and reinforcement of the responsible lesion of the diaphragm are performed, and these techniques are used either singly or in combination. In 1996, Di Bisceglie et al. reported the first case of VATS for PPC [7]. Observing the thoracic cavity more precisely, VATS can be a highly useful approach than thoracotomy. Saito et al. reviewed 29 cases of PPC and found that the overall treatment success rate for PPC is 72%. According to the previous report, the success rate is 89% in 21 fistula-confirmed cases, but only 38% in 8 cases in which fistulae are unconfirmed. In this case, by carefully inspecting the diaphragm with a thoracoscope, a hole was detected at the right central tendon of the diaphragm, and direct suturing was performed successfully.

In previous reports, PGA sheets, fibrin glue, and pericardial fat pad tissues are used to reinforce the diaphragm for treating PPC [8, 9]. However, the recurrence of PPC is still reported. In this case, reinforcement was strengthened for reducing the recurrence of hydrothorax more. As additional reinforcement materials, the latissimus dorsi and intercostal muscles can be considered. There is no report on the reinforcement of the diaphragm with pedicled muscle flaps. In this case, we selected a harvested pedicled LDM flap by minimal invasive technique, because it has a sufficient volume and blood flow for reinforcing the closed fistulae and its surroundings. Furthermore, for preventing the recurrence of hydrothorax from other parts of the central tendon center, we have used PGA sheet and fibrin glue.

## Conclusion

VATS was an effective method for treating PPC, and not only suturing the fistula but also reinforcing the responsible lesion of the diaphragm for preventing a recurrence of hydrothorax.

## Abbreviations

^99m^Tc-MAA: ^99m^Tc-macro-aggregated albumin
CAPD: Continuous ambulatory peritoneal dialysis
LDM: latissimus dorsi muscle
PGA: polyglycolic-acid
PPC: Pleuroperitoneal communication
VATS: Video-assisted thoracic surgery

## References

[1] Nomoto Y, Suga T, Nakajima K, Sakai H, Osawa G, Ota K (1989). Acute hydrothorax in continuous ambulatory peritoneal dialysis–a collaborative study of 161 centers. Am J Nephrol.

[2] Shishido T, Ryuzaki M, Takimoto C, Kobayashi E, Handa M, Yamamoto T (2010). A case of recurrent hydrothorax complicating continuous ambulatory peritoneal dialysis after video-assisted thoracoscopic surgery. J Jpn Soc Dial Ther.

[3] Chow KM, Szeto CC, Li PK (2003). Management options for hydrothorax complicating peritoneal dialysis. Semin Dial.

[4] Edwards SR, Unger AM (1967). Acute hydrothorax—a newcomplication of peritoneal dialysis. JAMA.

[5] Yim AP, Lee TW, Wan IY, Ng C (2002). Images in cardiothoracic surgery. Pleuroperitoneal fistula. Ann Thorac Surg.

[6] Saillen P, Mosimann F, Wauters JP (1991). Hydrothorax and end-stage chronic renal failure. Chest.

[7] Di Bisceglie M, Paladini P, Voltolini L, Garosi G, Ghiribelli C, Di paolo N (1996). Videothoracoscopic obliteration of pleuroperitoneal fistula in continuous ambulatory peritoneal dialysis. Ann Thorac Surg.

[8] Saito M, Nakagawa T, Tokunaga Y, Kondo T (2012). Thoracoscopic surgical treatment for pleuroperitoneal communication. Interact Cardiovasc Thorac Surg.

[9] Shoji F, Katsura M, Haratake N, Akamine T, Takamori S, Takada K (2017). Surgical repair of pleuroperitoneal communication with continuous ambulatory peritoneal dialysis. Thorac Cardiovasc Surg.

